# Case for diagnosis. Penile lesion in HIV-negative patient^☆^^☆☆^

**DOI:** 10.1016/j.abd.2020.03.006

**Published:** 2020-06-26

**Authors:** Mauro Cunha Ramos, Fabiana Bazanella de Oliveira, Vicente Sperb Antonello, Ana Leticia Boff

**Affiliations:** a Private Clinic, Porto Alegre, RS, Brazil; b Infection Control Service, Hospital Femina, Porto Alegre, RS, Brazil; c Dermatology Service, Santa Casa de Misericórdia de Porto Alegre, Porto Alegre, RS, Brazil

**Keywords:** Herpesvirus 8, human, Penile neoplasms, Kaposi's sarcoma

## Abstract

We present the case of an HIV-negative man with asymptomatic penile erythematoviolaceous papules associated with similar slightly verrucous papules in the interdigital space of the right foot. A biopsy of the penile lesion confirmed Kaposi's sarcoma. No other causes of immunosuppression were observed. Penile lesions of KS are rare in HIV-negative individuals but it should also be considered in the differential diagnosis. Careful follow-up is recommended.

## Case report

A middle-aged heterosexual Caucasian man of Italian origin was seen at a dermatology practice with asymptomatic lesions on the penis and without general symptoms. He reported having a regular sexual partner for several years, as well as occasional partners, with whom he reported regularly using condoms. He had been adequately treated for syphilis five years earlier and had no other significant health problems.

**Figure 1:**
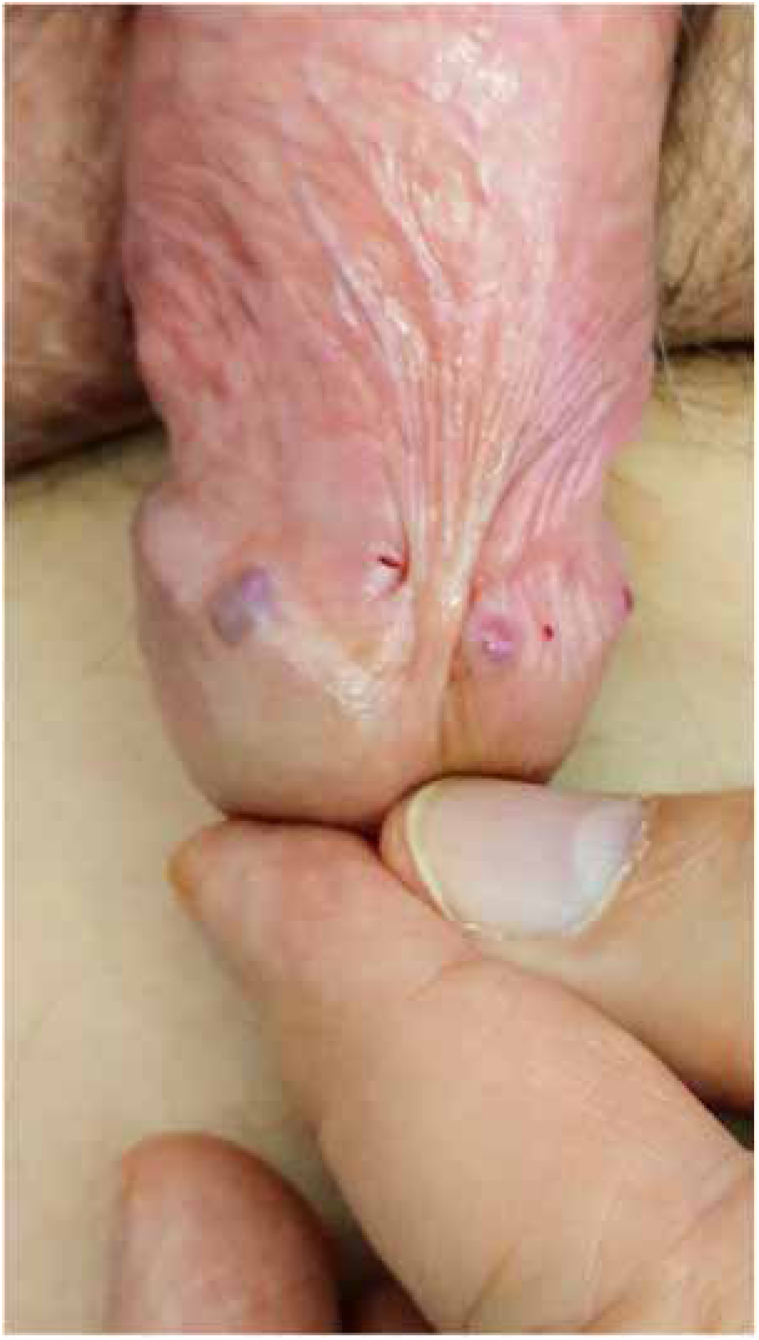
Erythematoviolaceous papules on the corona of the glans.

**Figure 2:**
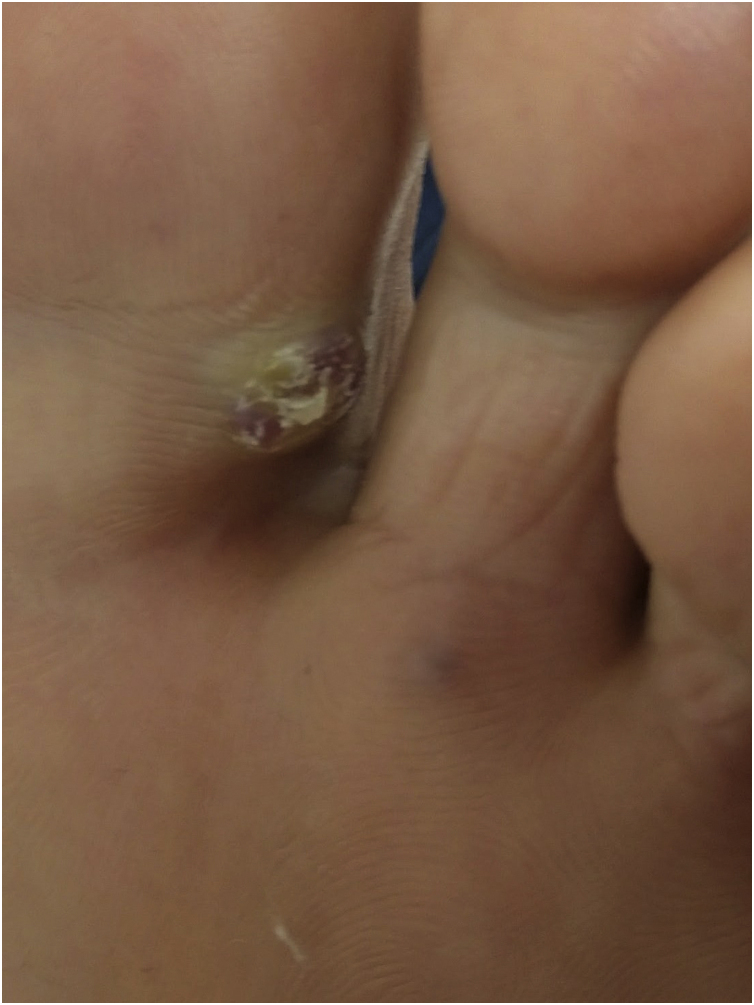
Violaceous papule with slightly verrucous surface in the interdigital space of the foot.

**Figure 3:**
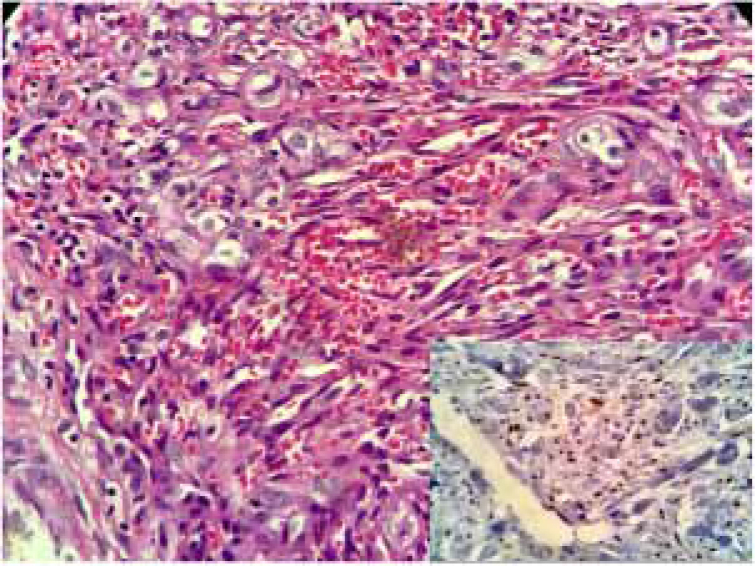
Histopathology revealed a fusocellular proliferation delineating elongated and branched vessels, extravasated red blood cells, frequent mitoses, and few cellular atypias. The immunohistochemical study in the inset.

Physical examination indicated erythematoviolaceous papules on the coronal margin of the glans on the ventral surface of the penis (Fig. 1). A general examination of the skin evidenced similar lesions in the first interdigital space of the right foot, one of which was slightly verrucous (Fig. 2). The histopathological study of one of the penile lesions revealed a fusocellular proliferation with elongated and branched vessels, hemorrhage, frequent mitoses, and few cellular atypias (Fig. 3).

## What is your diagnosis?

a) Molluscum contagiosum
b) Genital warts
c) Kaposi's sarcoma
d) Pyogenic granuloma

In greater detail, gaps with extravasated erythrocytes, few atypical cells, and several mitoses can be observed (Fig. 3). The immunohistochemical study detected nuclear positivity for antibodies against the Kaposi's sarcoma-associated herpesvirus (Fig. 3, inset). Contrary to clinical expectations, fourth-generation ELISA tests, performed at a two-month interval, were negative on both occasions. To better discard HIV infection, viral load was measured by a PCR test, which was also negative. Serology for hepatitis B and C was negative; FTA-Abs was positive and VDRL was non-reactive. Computed tomography of chest and abdomen did not show any lesions. Cryotherapy sessions were unsuccessful. The lesions were surgically excised; the recurrence of one small lesion was observed after eight months of follow-up. As no additional manifestations were observed, a new excision was performed and a strict follow-up was recommended.

## Discussion

Kaposi's sarcoma (KS) is a spindle cell neoplasm described in 1872 by Moritz Kaposi. It is a rare disease that is usually observed in men over 60 years of age, especially of Mediterranean and/or Jewish origin. It commonly appears in the lower limbs, in which it remains indolent. Metastases and deaths are rarely reported.^1^ Three other forms have been described. The aggressive endemic form is found in Africa and was first described in 1914. The iatrogenic form affects transplant recipients (mainly in cases of solid organ transplants and immunosuppressed patients), especially those who use corticosteroids for long periods. The AIDS-related form (epidemic form) was identified among men who had sex with men in the early 1980s.^2^ In 1990, through evaluations of epidemiological information, it was inferred that the etiologic agent of KS would be a sexually transmitted virus, which was confirmed in 1994 by Chang et al.^3^ This form of the disease has a variable course, but it can be very aggressive, leading to spread and death. Lesions on the genitals and/or the oral cavity are usually identified as an initial manifestation of AIDS. The use of antiretroviral therapy has reduced its global incidence and often reverses the course of KS.^4^

The Kaposi's sarcoma-associated herpesvirus is a gammaherpesvirus (HHV-8). Infection is considered necessary, but not sufficient, for the development of this sarcoma. Its transmission through saliva or other body fluids is accompanied by an unpredictable period of latency that is similar in other herpesviruses.^5^ In endemic regions, such as Africa, over 80% of adolescents may have antibodies to the virus. In the general population of northern Europe and the United States, the serum prevalence is less than 5%; however, in homosexual or bisexual men, a serum prevalence of up to 25% has been described. In individuals with normal immunity, its presence rarely leads to KS.

Penile lesions of KS are rare in HIV-negative heterosexual individuals, which highlights the relevance of the present case report. Few cases of KS of the penis among patients without HIV infection have been published in the literature.^6^ The authors’ initial belief was that the patient had an otherwise asymptomatic infection, an assumption that was ruled out after laboratory investigation. Despite the need for a high level of suspicion regarding HIV infection when assessing patients with KS penile lesions, careful pre-test counseling is required to avoid jumping to conclusions about the diagnosis of HIV infection and its emotional consequences.

## Financial support

None declared.

## Authors' contributions

Mauro Cunha Ramos: Approval of the final version of the manuscript; elaboration and writing of the manuscript; intellectual participation in propaedeutic and/or therapeutic conduct of studied cases; critical review of the literature; critical review of the manuscript.

Fabiana Bazanella de Oliveira: Elaboration and writing of the manuscript; obtaining, analyzing, and interpreting the data; critical review of the literature; critical review of the manuscript.

Vicente Sperb Antonello: Approval of the final version of the manuscript; intellectual participation in propaedeutic and/or therapeutic conduct of studied cases; critical review of the literature.

Ana Leticia Boff: Approval of the final version of the manuscript; intellectual participation in propaedeutic and/or therapeutic conduct of studied cases.

## Conflicts of interest

None declared.

## References

[1] Ohe E.M.D.N., Enokihara M.M.S.S., Porro A.M., de Q Padilha M.H.V., de Almeida F.A. (2010). Fatal outcome in classic Kaposi's sarcoma. An Bras Dermatol.

[2] Hymes K.B., Greene J.B., Marcus A., William D.C., Cheung T., Prose N. (1981). Kaposi's sarcoma in homosexual men – a report of eight cases. Lancet.

[3] Newton R., Whitby D. (2016). Beral et al.'s 1990 paper on Kaposi's sarcoma among persons with AIDS: demonstrating the power of descriptive epidemiology. Cancer Epidemiol.

[4] Semeere A.S., Busakhala N., Martin J.N. (2012). Impact of antiretroviral therapy on the incidence of Kaposi's sarcoma in resource-rich and resource-limited settings. Curr Opin Oncol.

[5] Dittmer D.P., Damania B. (2016). Kaposi sarcoma-associated herpesvirus: immunobiology, oncogenesis, and therapy. J Clin Invest.

[6] Attwa E., Gharib K., Albalat W., Amer A. (2016). Classical Kaposi sarcoma: case reports with unusual presentation on the penis and scrotum. Int J Dermatol.

